# Autonomic disorders and myocardial 123I-metaiodobenzylguanidine scintigraphy in Huntington’s disease

**DOI:** 10.1007/s12350-020-02299-7

**Published:** 2020-08-16

**Authors:** Roberta Assante, Elena Salvatore, Carmela Nappi, Silvio Peluso, Giovanni De Simini, Luigi Di Maio, Gianluigi Rosario Palmieri, Isabella Pia Ferrara, Alessandro Roca, Giuseppe De Michele, Alberto Cuocolo, Sabina Pappatà, Anna De Rosa

**Affiliations:** 1 Department of Advanced Biomedical Sciences, Federico II University, Naples, Italy; 2 Department of Neurosciences and Reproductive and Odontostomatological Sciences, Federico II University, Via Pansini 5, 80131 Naples, Italy; 3 Institute of Biostructure and Bioimaging, National Council of Research, Naples, Italy

**Keywords:** Huntington’s disease, autonomic disorders, scintigraphy, MIBG

## Abstract

**Background:**

Huntington’s disease (HD) patients often present with abnormal modulation of blood pressure and heart rate. We investigated whether cardiac autonomic innervation assessed by 123I-metaiodobenzylguanidine (MIBG) imaging is impaired in HD patients, in comparison with controls (Ctrl).

**Methods:**

Fifteen patients (6 F and 9 M) were assessed by the motor section of the Unified HD Rating Scale, the Total Function Capacity, and the scale for outcomes in Parkinson’s disease-autonomic (SCOPA-AUT) questionnaire. All patients and 10 Ctrl (5 F and 5 M) underwent 123I-MIBG imaging. From planar images, the early and late heart-to-mediastinum (H/M) ratios and myocardial washout rates (WR) were calculated.

**Results:**

We did not find significant differences in early and late H/M ratios and WR between the two groups. At individual level, three patients showed reduced early and/or late H/M ratios. The most common autonomic complaints were gastrointestinal and genitourinary disorders. SCOPA-AUT questionnaire score results positively correlated with the disease duration and WR.

**Conclusions:**

Our study indicates that myocardial postganglionic sympathetic innervation is essentially preserved or only minimally involved in HD. These findings suggest that the cardiovascular dysfunction might be mainly due to the impairment of brain areas associated with the regulation and modulation of the heart function.

**Electronic supplementary material:**

The online version of this article (10.1007/s12350-020-02299-7) contains supplementary material, which is available to authorized users.

## Introduction

Huntington’s disease (HD) is an autosomal dominant neurodegenerative disorder caused by a CAG trinucleotide repeat expansion in the huntingtin gene on chromosome 4 which leads to the production of a protein with an abnormally long polyglutamine stretch.^1^ The prevalence is 10.6 to 13.7 × 10^−5^ in Western countries and the age at onset ranges between 30 and 50 years.^1^,^2^ The cardinal symptoms consist of movement disorders, usually chorea, cognitive impairment, and psychiatric disturbances.^1^,^2^

The clinical picture is also characterized by symptoms due to hypothalamic dysfunction as weight loss, sleep and endocrine disorders including increased cortisol levels, reduced testosterone levels and high prevalence of diabetes.^3^ The patients may complain of autonomic disorders that often precede the onset of motor manifestations. HD patients and pre-manifest CAG expansion carriers more significantly present with gastrointestinal, urinary, cardiovascular and, in men, sexual problems than the control subjects.^4^ In particular, potential cardiac manifestation of severe autonomic dysfunction, as light headedness on standing up, tachycardia, arrhythmias, and sudden cardiac death, seems to be more frequent in HD than in controls.^4^,^5^

Here, we aimed to investigate whether cardiac autonomic innervation assessed by 123I-metaiodobenzylguanidine (MIBG) imaging is impaired in HD patients, in comparison with control subjects (Ctrl).

## Patients and Methods

We included fifteen HD patients (6 F and 9 M), with confirmation by genetic test, and ten Ctrl (5 F and 5 M), comparable for age. Written informed consent was obtained from all participants, according to the declaration of Helsinki and with the local Ethics Committee approval. Patients were assessed by the motor examination of the Unified HD Rating Scale (UHDRS, section III), and the Total Function Capacity (TFC). All patients underwent the Scale for Outcomes in Parkinson’s Disease-Autonomic (SCOPA-AUT) questionnaire,^6^ to assess self-reported autonomic dysfunction. Subjects with MMSE score ≤ 23/30, cardiac disease, diabetes, untreated hypertension or receiving treatment with tricyclic and tetracyclic antidepressants, serotonin reuptake inhibitors (SSRI), sympathomimetics and sympatholytics, antipsychotics, calcium channel antagonists or ACE inhibitors were excluded, to avoid interference with MIBG uptake.^7^ Ten subjects served as the Ctrl group, undergoing 123I-MIBG scintigraphy to rule out disease of the adrenal medulla. None of these subjects had a history of neurological or cardiac diseases or diabetes and none of them was taking medication that might have been expected to interfere with MIBG myocardial uptake. All patients and Ctrl underwent 123I-MIBG cardiac imaging as previously described in detail.^8^ After an administration of 111 MBq of 123I-MIBG a 10-minute planar anterior chest image (256x256 matrix) was performed at 15 minutes (‘‘early’’ image) and again at 3 hours and 50 minutes (‘‘late’’ image). From planar images of the thorax, the early and late heart-to-mediastinum (H/M) ratios were computed by dividing the mean counts per pixel within the myocardium by the mean counts per pixel within the mediastinum. By comparing early and late activities, the 123I-MIBG washout rate (WR) from the myocardium was derived, providing a parameter that reflects retention of norepinephrine by sympathetic neurons. The MIBG WR was calculated using the formula:

$$ \frac{{\left[ {\left( {{\text{early}}\;{\text{heart}}\;{\text{counts}}\;{\text{per}}\;{\text{pixel}}{-}{\text{early}}\;{\text{mediastinum}}\;{\text{counts}}\;{\text{per}}\;{\text{pixel}}} \right) - \left( {{\text{late}}\;{\text{heart}}\;{\text{counts}}\;{\text{per}}\;{\text{pixel}}\;{\text{decay}}\;{\text{corrected}} - {\text{late}}\;{\text{mediastinum}}\;{\text{counts}}\;{\text{per}}\;{\text{pixel}}\;{\text{decay}}\;{\text{corrected}}} \right)} \right]}}{{\left( {{\text{early}}\;{\text{heart}}\;{\text{counts}}\;{\text{per}}\;{\text{pixel}} - {\text{early}}\;{\text{mediastinum}}\;{\text{counts}}\;{\text{per}}\;{\text{pixel}}} \right)}} \times 100 $$

### Statistical Analysis

Differences in non-parametric data between HD patients and Ctrl were analyzed using the Mann–Whitney *U* test. The relationship between variables was examined using the Spearman’s correlation coefficient. A *P* value < 0.05 was considered statistically significant. The Statistical Package for the Social Sciences software for Windows (version 21.00, SPSS, Chicago, IL, USA) was used for the statistical analyses.

## Results

Demographic and clinical characteristics and imaging findings are shown in Table 1. Ten patients were in stage 1 of disease (TFC 11 to 13), and 5 in stage 2 (TFC 7 to 10). At SCOPA-AUT questionnaire, dysphagia was reported by 9 subjects (64%; case 1, 4, 5, 6, 8, 11, 13, 14 and 15), drooling by 3 (20%; case 10, 13 and 14), gastrointestinal symptoms, as feeling that food gets stuck in throat or constipation, by 9 patients (60%; case 1, 6, 7, 8, 9, 10, 11, 13 and 14), genitourinary symptoms, as bladder and/or sexual dysfunction, by 10 (67%; case 1, 2, 4, 5, 7, 9, 10, 11, 13 and 14), symptoms related to orthostatic hypotension, as the feeling of either becoming lightheaded when suddenly standing up or after standing for a while, by 6 (40%; case 1, 7, 9, 10, 12 and 13), sweating dysfunction by 6 (40%; case 1, 4, 7, 10, 12 and 15), oversensitivity to bright light and trouble tolerating cold/heat both by 7 (47%; case 4, 7, 10, 11, 12, 13, 14; case 1, 4, 7, 10, 12, 13 and 15) (Table 2).

**Table 1:** Demographic and clinical data and MIBG scintigraphy results

Subjects	Gender	Age	DD	SCOPA-AUT	UHDRS-III	TFC	CAG	Early H/M	Late H/M	WR
Patients										
1	F	50	10	15	20	13	43	2.18	2.04	29.27
2	M	51	1	5	27	11	44	**1.48**	**1.62**	7.31
3	M	65	3	10	25	11	40	**1.7**	**1.5**	39.0
4	F	68	5	10	33	9	41	2.5	2.7	11.5
5	M	47	5	13	34	10	46	1.96	1.9	25.66
6	F	64	8	7	35	11	43	2.06	2.17	16.7
7	F	41	4	26	15	10	44	2.28	1.82	25.6
8	F	39	3	4	11	13	48	**1.88**	1.85	15.0
9	M	46	2	0	4	13	42	1.95	2.05	18.5
10	M	66	11	22	42	7	42	2.02	1.98	37.5
11	F	74	3	14	11	13	40	2.3	2.2	19.21
12	M	35	1	6	8	13	47	2.31	2.45	-6.55
13	M	47	6	10	12	12	44	2.05	2.59	3.64
14	M	42	7	12	45	8	49	2.32	2.10	39.0
15	M	55	4	8	22	11	45	2	2.2	1.35
Mean ± SD		52.6 ± 12.0	4.8 ± 3.0	10.8 ± 9.0	24.6 ± 12.0			2.06 ± 0.26	2.09 ± 0.35	18.84 ± 14.01
Median						11	43			
Ctrl		55.0 ± 7.0								
Mean ± SD								2.18 ± 0.11	2.11 ± 0.18	20.65 ± 9.28

*DD*, disease duration; *SCOPA-AUT*, Scale for outcomes in Parkinson’s disease-autonomic; *UHDRS*, section III of Unified Huntington’s Disease Rating Scale; *TFC*, total functional capacity; *CAG*, pathological repeat

Values below or above 2SD the mean of Ctrl were considered abnormal (in bold)

**Table 2:** Prevalence of autonomic complaints at SCOPA-AUT questionnaire in 15 patients with Huntington’s disease.

	Patients (%)
Gastrointestinal domain	9 (60)
Genitourinary domain	10 (67)
Cardiovascular domain	6 (40)
Thermoregulatory domain	7 (47)
Pupillomotor domain	7 (47)

**Figure 1:**
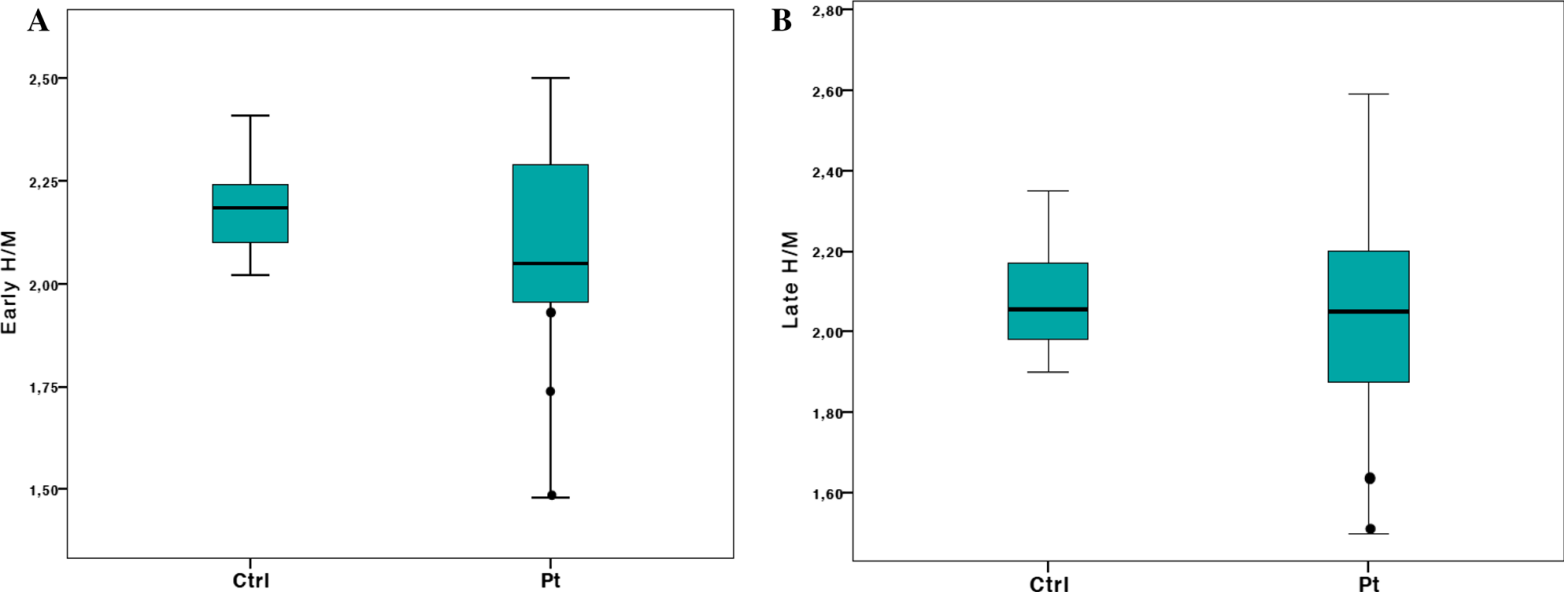
Early H/M (A) and late H/M ratio (B) results in patients (Pt) and controls (Ctrl). The central boxes represent the values from the lower to upper quartile (25° to 75° percentile). The middle lines represent the mean. The vertical lines extend from the minimum to the maximum value; pathological values are displayed as separate points

There were no significant differences in early H/M ratio (*P* = 0.18; cut-off ≥ 1.96), late H/M ratio (*P* = 0.85; cut-off ≥ 1.75) and WR (*P* = 0.78; cut-off ≤ 39.4%) between HD patients and Ctrl (Figure 1). At the individual level, compared to Ctrl, abnormal results (2SD below or above the mean of Ctrl) were found in only three patients: two subjects showed a significant reduction in both early and late H/M ratios (case 2 and 3) and one in early H/M ratio only (case 8) (Table 1).

As expected, a correlation was observed between CAG expansion and age at onset (rho − 0.598; *P* < 0.02), and between disease duration and UHDRS-III score (rho 0.635; *P* = 0.011).

**Figure 2:**
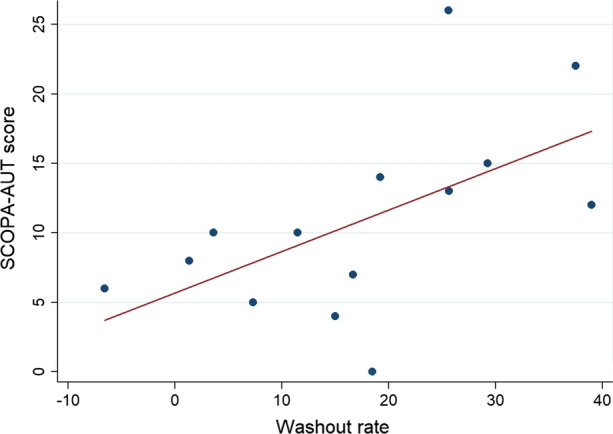
Relationship between SCOPA-AUT score and washout rate (Spearman’s correlation; rho 0.631; *P* = 0.015)

SCOPA-AUT questionnaire score results positively correlated with both disease duration (rho 0.598; *P* = 0.024) and WR (rho 0.631; *P* = 0.015) (Figure 2).

## Discussion

In the last years, the evidence of an autonomic dysfunction in HD, even in presymptomatic and early stage of the disease, has strengthened. Abnormal modulation of blood pressure and heart rate, manifesting with syncope and cardiac arrhythmias, often occur in HD. Abnormal circadian rhythm of heart rate, decreased heart rate variability and baroreceptor reflex failure have been observed in preclinical and clinical research.^9^^–^11 Recent studies showed the presence of both sympathetic and parasympathetic autonomic nervous system dysfunction in HD.^10^^–^12

In a previous study in patients affected by spinocerebellar ataxia type 2, another degenerative disorder due to CAG triplet expansion and associated with abnormal aggregates of polyglutamine sequences, 123I-MIBG myocardial scintigraphy showed an impairment of cardiac sympathetic function.^13^

In view of the common pathogenic mechanism underlying the two disorders, we also assessed self-reported autonomic complaints and 123I-MIBG imaging in HD patients, to investigate if postganglionic sympathetic innervation is impaired in this neurodegenerative disorder, as so far this tool has never been applied in HD.

Autonomic dysfunction was present in our HD patients. Swallowing difficulties, erection and ejaculation problems, dysphagia, and drooling were the most common autonomic symptoms found in our patients in comparison with Ctrl using the SCOPA-AUT questionnaire, in agreement with previous report.^4^ We also found a high prevalence of gastrointestinal and genitourinary symptoms, but sweating dysfunction, symptoms related to orthostatic hypotension, oversensitivity to bright light and trouble tolerating cold/heat also resulted common. We observed a significant association between the total autonomic dysfunction score and the disease duration, suggesting that the autonomic nervous system involvement worsens over the time.

Interestingly, the SCOPA-AUT questionnaire score was not correlated with CAG expansion size, UHDRS-III score, and TFC, suggesting that the autonomic dysfunction could be an early non-motor manifestation of disease, in line with previous findings.^4^

The finding of oversensitivity to bright light might be due to retinal dysfunction/degeneration reported in mouse models and patients with HD. Attenuated pupillary light response accompanied by a progressive downregulation of retinal cone opsin and melanopsin expression has been observed in mouse models.^14^ The pupillary light reflex latency has been shown increased in the HD group in comparison with Ctrl.^15^

Thermoregulatory disorders as hypothermia, erratic thermogenesis, and an abnormal circadian temperature rhythm have already been reported in HD,^16^ so the trouble tolerating cold/heat complained by our patients may be explained by both metabolic factors, as low body weight, energy expenditure due to dyskinesias and adipose tissue composition, and hypothalamic pathology characterized by oxytocin and vasopressin neuron loss.^16^

We did not find a significant difference in early and late H/M ratios and WR between HD patients and Ctrl as a group (Table 1). At the individual level, two patients with HD showed, however, a significant reduction in both early and late H/M ratios, and one in early H/M only. This finding is not easy to interpret as the disease was at an early stage in all three patients. Moreover, those patients did not differ from the others in severity of the clinical and genetic features (age at onset, motor UHDRS score, CAG expansion, disease duration, age, SCOPA-AUT scores) (Table 1), and the presence of autonomic cardiovascular symptoms. The cerebral mechanisms for control of the autonomic nervous system are complex and not yet well understood.^5^ The wide and heterogeneous neuropathological findings reported in HD^17^,^18^ may explain the heterogeneity of both results in evaluation of cardiac sympathetic activity and of autonomic disorders detected at SCOPA-AUT questionnaire.

Interestingly, we observed a significant association between SCOPA-AUT questionnaire score and WR, that accounts for the retention of norepinephrine by sympathetic neurons and overall sympathetic tone, mainly representing noradrenaline uptake-1 (Figure 2).^19^ This finding is consistent with a relevant contribution of sympathetic impairment to the autonomic dysfunction in our patients.

Overall, the results of our study suggest that myocardial postganglionic sympathetic innervation as measure in vivo with 123I-MIBG is mostly preserved or only slightly reduced in HD, as suggested by the occurrence of significant early and/or late H/M ratios reduction in 20% of patients and the correlation found between increased WR and the severity of autonomic dysfunction.

The impairment of cortical and subcortical regions, as the prefrontal cortex, the bilateral insular cortex, the anterior cingulate gyrus, the amygdala and the hypothalamus, often reported in HD^20^ and implicated in the regulation and control of the cardiac function, might partly underly the cardiovascular dysfunction found in our HD patients.

Brain-derived neurotrophic factor (BDNF) is known to be involved in the neuro-mediated regulation of heart rate and blood pressure and to play a protective role against cardiac dysfunction.^21^ BDNF and its receptor TrkB are highly expressed in areas involved in cardiac control regions such as amygdala, frontal cortex, hypothalamus, and the brainstem. Interestingly, decreased levels of BDNF are reported in the striatum, cortex, and brainstem of HD patients.^21^ A deficient cortical transcription of the BDNF gene, its defective transport to the striatum and a reduced level of mRNA coding TrkB in the caudate nucleus have been also shown.^21^ Moreover, the accumulation of mutated huntingtin may be toxic to cardiomyocytes in humans, as showed in mouse models that developed cardiac dysfunction progressing to severe failure over a few weeks.^22^

So, cardiac dysfunction in HD is probably multifactorial and likely worsened by drugs, as neuroleptics and SSRI, that act on rhythm, heart rate and atrioventricular conduction.

### Limitations

Caution is required in the interpretation of our results as the number of subjects included in this study is relatively small. However, HD is a rare disease and 123I-MIBG scintigraphy requires strict criteria of inclusion to avoid drugs interfering with the analysis. Further investigation in a larger number of patients is required to confirm these findings. Despite this limitation, our work provides for the first time the status of cardiac sympathetic function in vivo in a cohort of HD patients.

### New Knowledge Gained

For the first time, 123I-MIBG has been performed in HD patients, showing that myocardial postganglionic sympathetic innervation is preserved in most cases. Therefore, the cardiovascular autonomic disorders may be due to central nervous system control impairment of the heart function.

## Electronic supplementary material

Below is the link to the electronic supplementary material.

Electronic supplementary material 1 (PPT 358 kb)

Electronic supplementary material 2 (M4A 2416 kb)

## Abbreviations

HD: Huntington’s disease
UHDRS: Unified HD Rating Scale
TFC: Total functional capacity
SCOPA-AUT: Scale for outcomes in Parkinson’s disease-autonomic
MIBG: Metaiodobenzylguanidine
H/M: Heart to mediastinum
WR: Washout rate
Ctrl: Control subjects
