# Supplementary material for: Autonomic disorders and myocardial 123I-metaiodobenzylguanidine scintigraphy in Huntington’s disease
Source: J Nucl Cardiol. 2020 Aug 16;29(2):642–8. doi: 10.1007/s12350-020-02299-7 (PMC8993714; doi:10.1007/s12350-020-02299-7)
Supplement: Supplementary file 1 — Electronic supplementary material 1 (PPT 358 kb) [file 12350_2020_2299_MOESM1_ESM.ppt]

## Slide 1
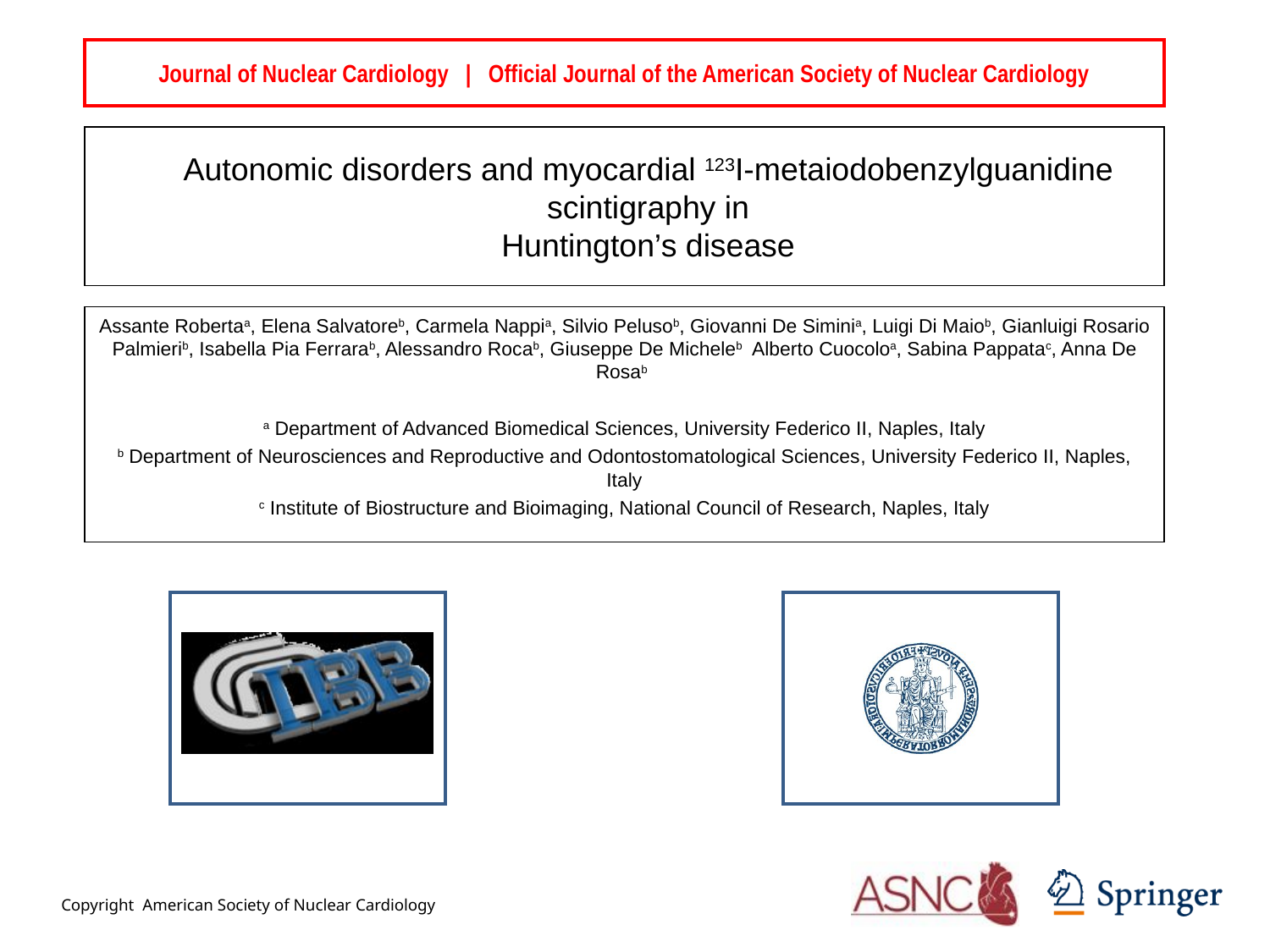

Journal of Nuclear Cardiology | Official Journal of the American Society of Nuclear Cardiology
# Autonomic disorders and myocardial 123I-metaiodobenzylguanidine scintigraphy in Huntington’s disease
Assante Robertaa, Elena Salvatoreb, Carmela Nappia, Silvio Pelusob, Giovanni De Siminia, Luigi Di Maiob, Gianluigi Rosario Palmierib, Isabella Pia Ferrarab, Alessandro Rocab, Giuseppe De Micheleb Alberto Cuocoloa, Sabina Pappatac, Anna De Rosab
a Department of Advanced Biomedical Sciences, University Federico II, Naples, Italy
b Department of Neurosciences and Reproductive and Odontostomatological Sciences, University Federico II, Naples, Italy
c Institute of Biostructure and Bioimaging, National Council of Research, Naples, Italy
Copyright American Society of Nuclear Cardiology

## Slide 2
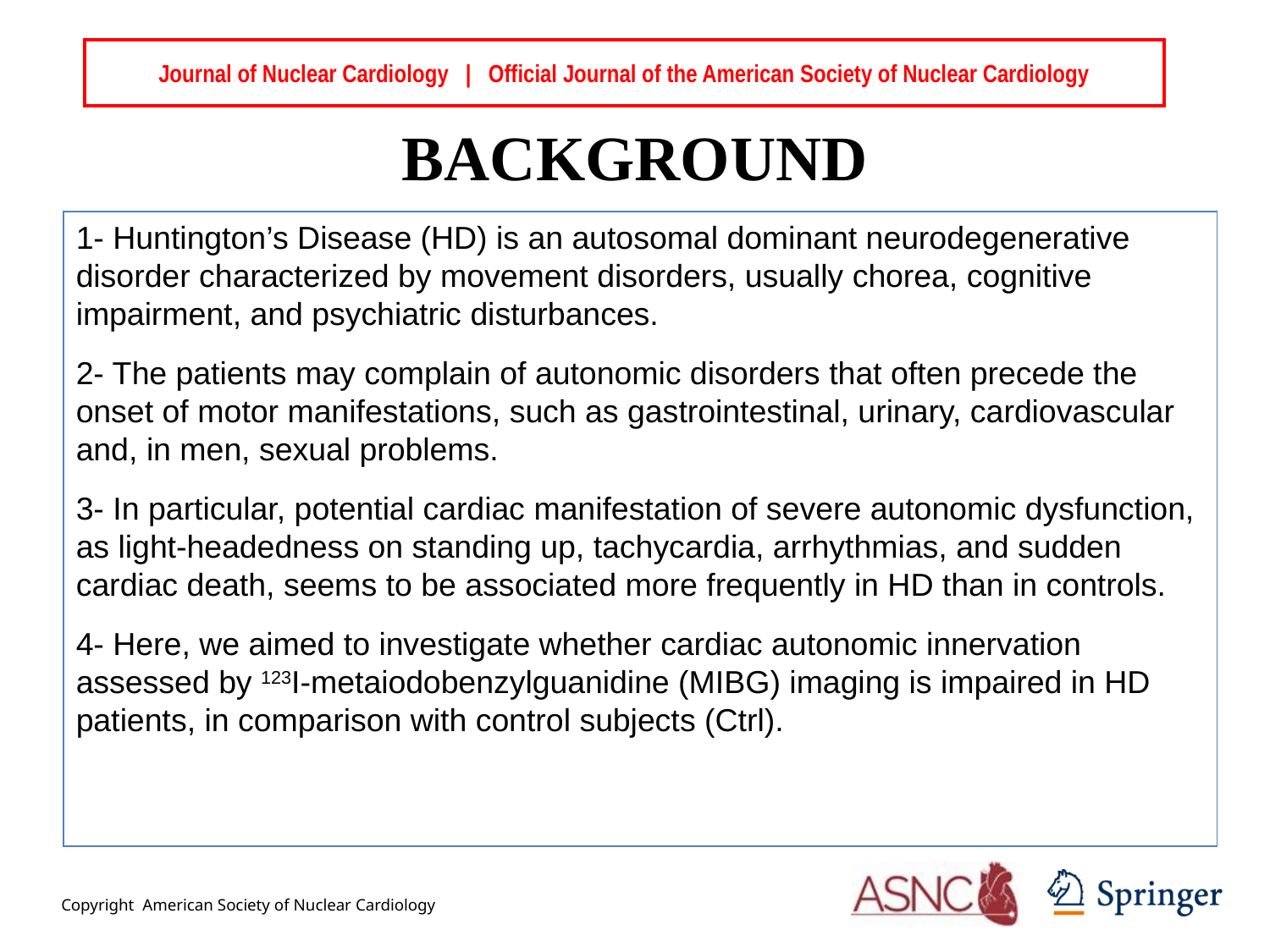

Journal of Nuclear Cardiology | Official Journal of the American Society of Nuclear Cardiology
# BACKGROUND
1- Huntington’s Disease (HD) is an autosomal dominant neurodegenerative disorder characterized by movement disorders, usually chorea, cognitive impairment, and psychiatric disturbances.
2- The patients may complain of autonomic disorders that often precede the onset of motor manifestations, such as gastrointestinal, urinary, cardiovascular and, in men, sexual problems.
3- In particular, potential cardiac manifestation of severe autonomic dysfunction, as light-headedness on standing up, tachycardia, arrhythmias, and sudden cardiac death, seems to be associated more frequently in HD than in controls.
4- Here, we aimed to investigate whether cardiac autonomic innervation assessed by 123I-metaiodobenzylguanidine (MIBG) imaging is impaired in HD patients, in comparison with control subjects (Ctrl).
Copyright American Society of Nuclear Cardiology

## Slide 3
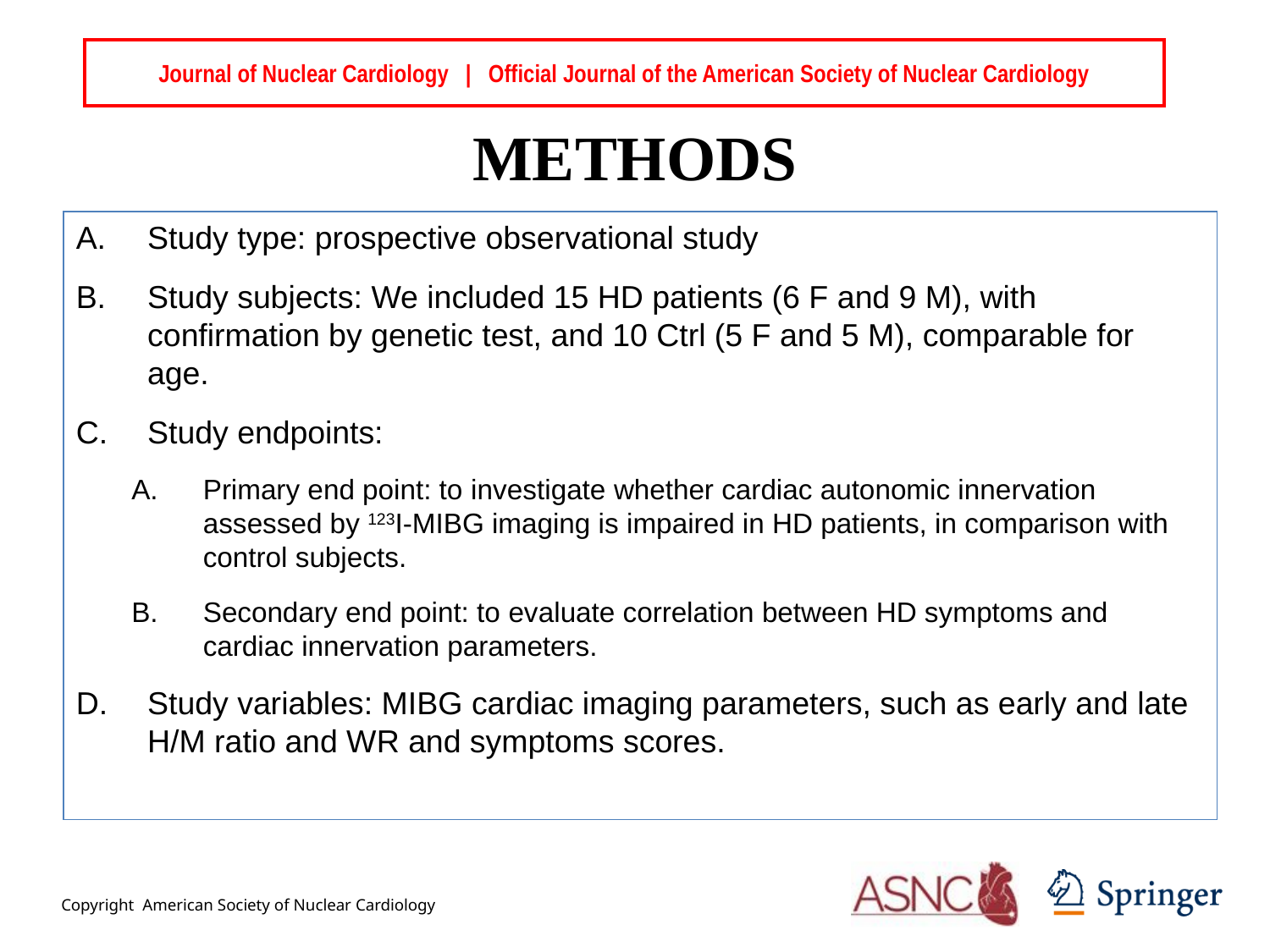

Journal of Nuclear Cardiology | Official Journal of the American Society of Nuclear Cardiology
# METHODS
Study type: prospective observational study
Study subjects: We included 15 HD patients (6 F and 9 M), with confirmation by genetic test, and 10 Ctrl (5 F and 5 M), comparable for age.
Study endpoints:
Primary end point: to investigate whether cardiac autonomic innervation assessed by 123I-MIBG imaging is impaired in HD patients, in comparison with control subjects.
Secondary end point: to evaluate correlation between HD symptoms and cardiac innervation parameters.
Study variables: MIBG cardiac imaging parameters, such as early and late H/M ratio and WR and symptoms scores.
Copyright American Society of Nuclear Cardiology

## Slide 4
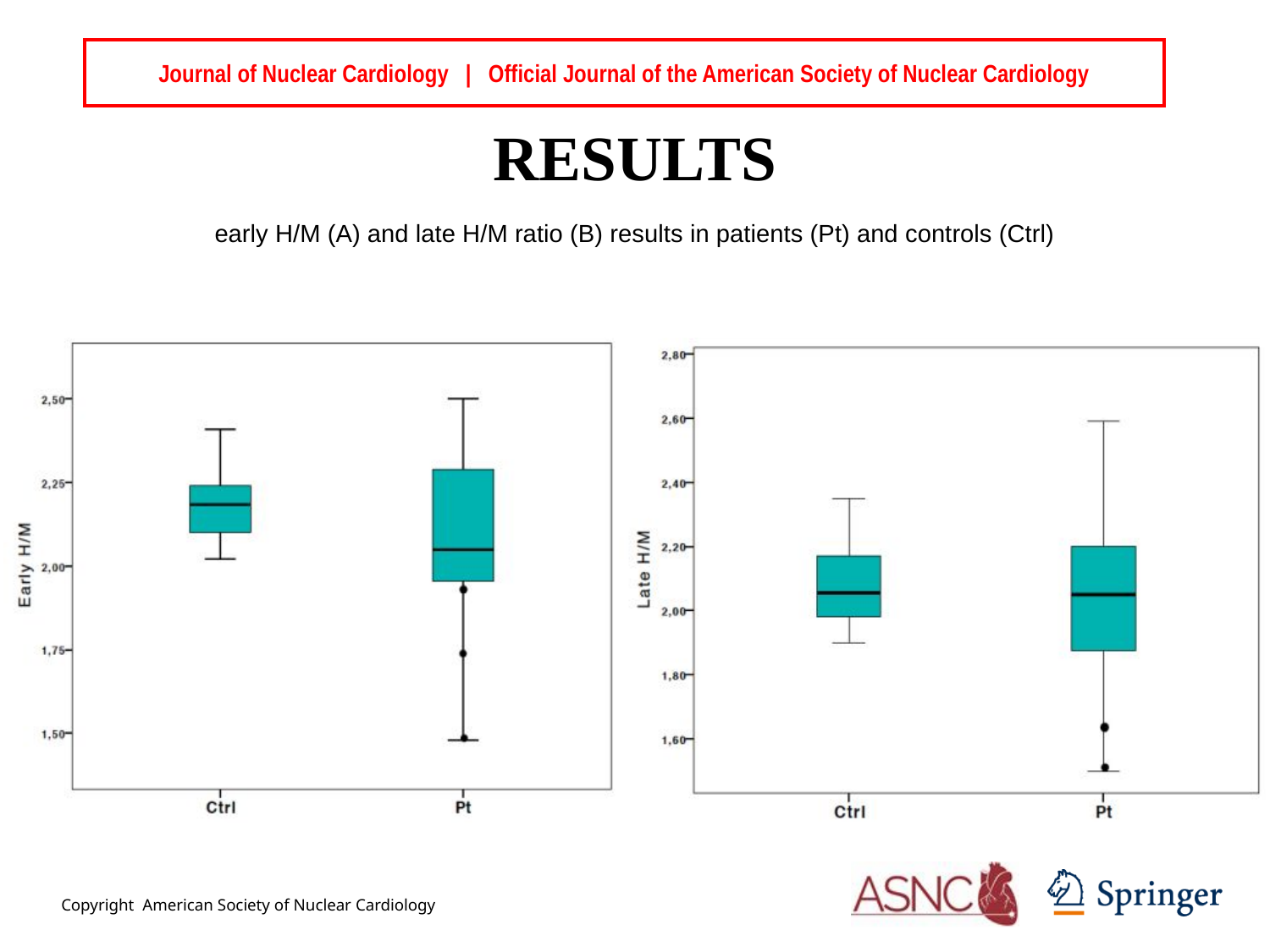

Journal of Nuclear Cardiology | Official Journal of the American Society of Nuclear Cardiology
# RESULTS
early H/M (A) and late H/M ratio (B) results in patients (Pt) and controls (Ctrl)
Copyright American Society of Nuclear Cardiology

## Slide 5
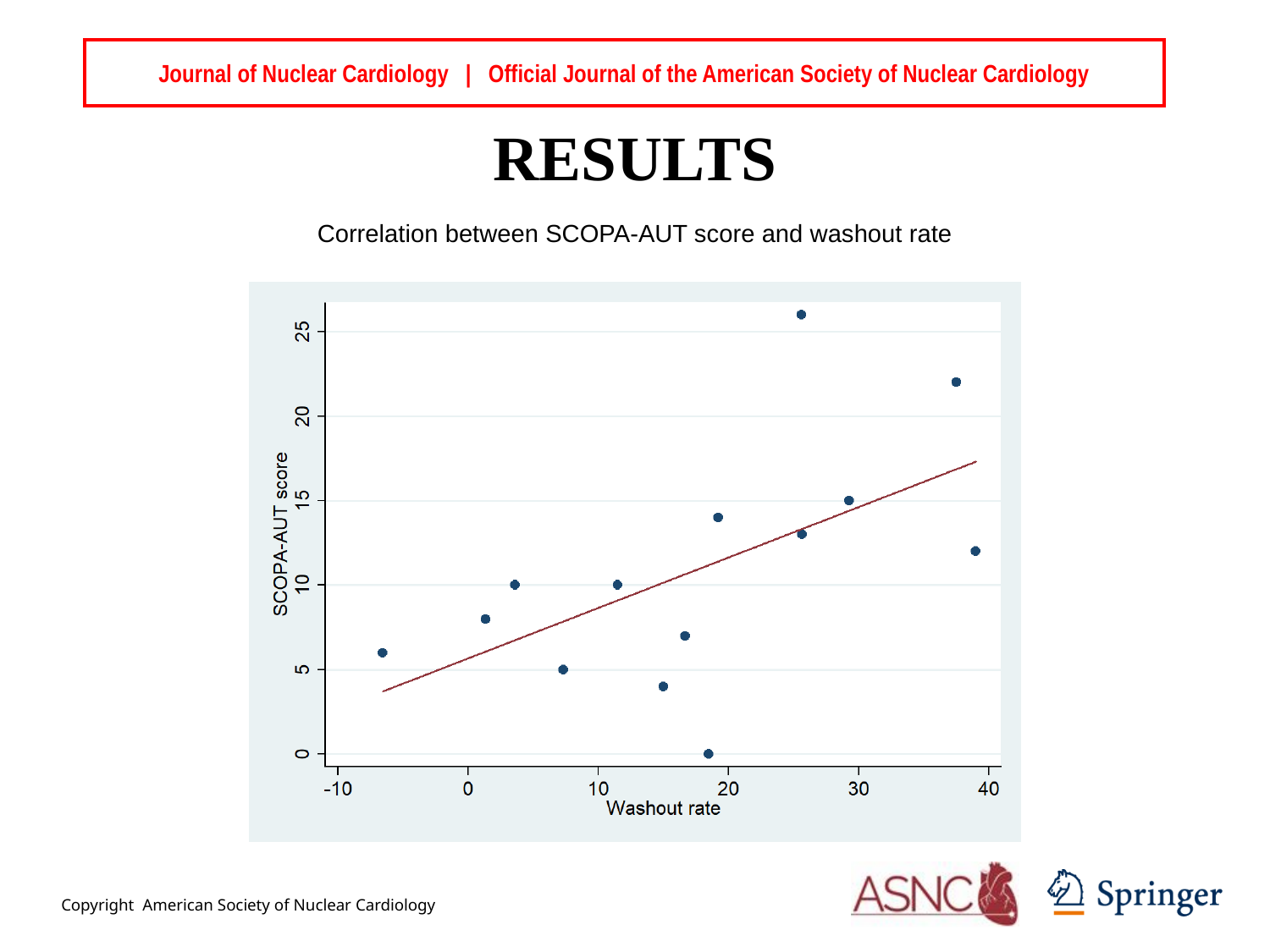

Journal of Nuclear Cardiology | Official Journal of the American Society of Nuclear Cardiology
# RESULTS
Correlation between SCOPA-AUT score and washout rate
Copyright American Society of Nuclear Cardiology

## Slide 6
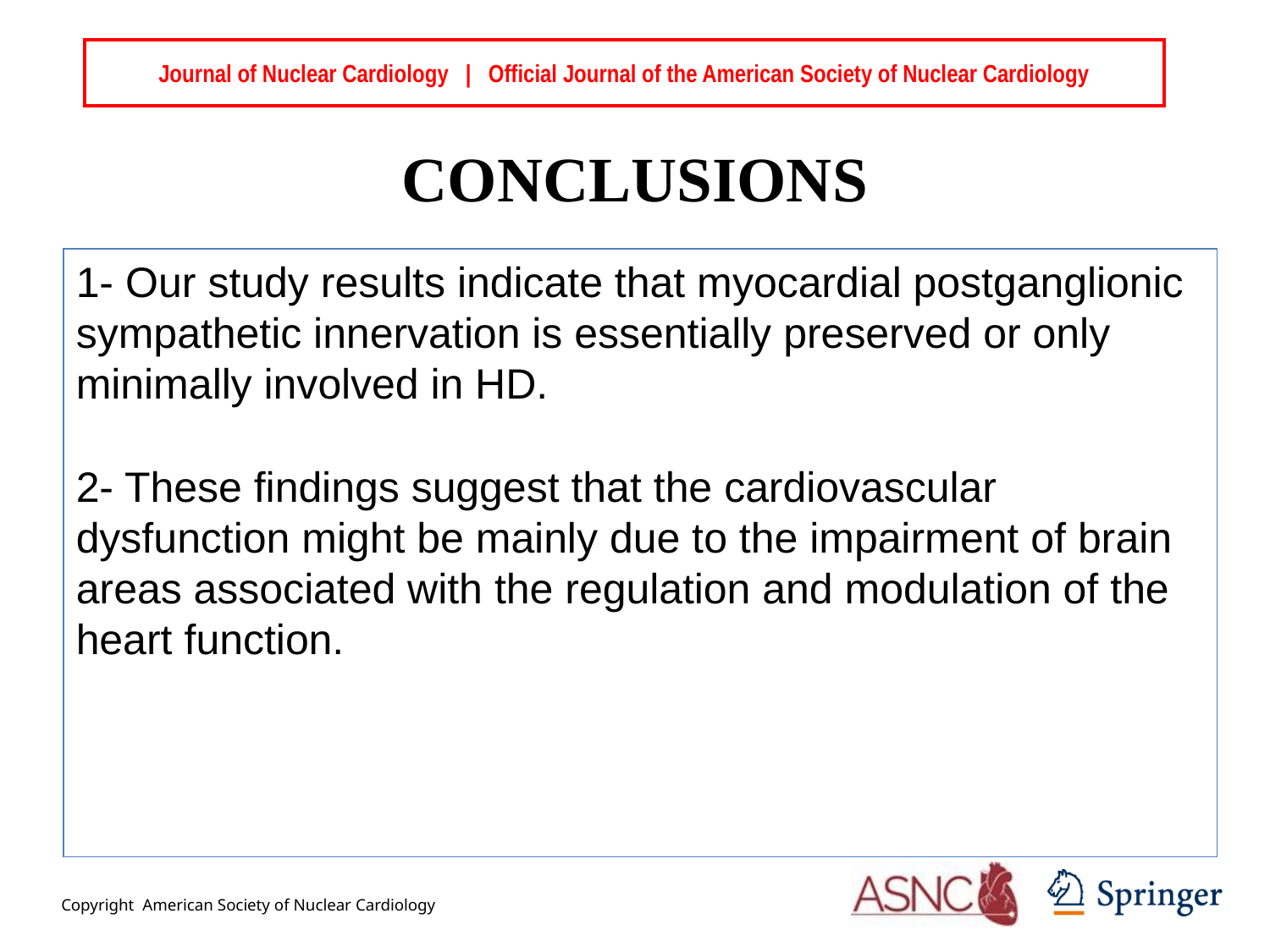

Journal of Nuclear Cardiology | Official Journal of the American Society of Nuclear Cardiology
# CONCLUSIONS
1- Our study results indicate that myocardial postganglionic sympathetic innervation is essentially preserved or only minimally involved in HD.
2- These findings suggest that the cardiovascular dysfunction might be mainly due to the impairment of brain areas associated with the regulation and modulation of the heart function.
Copyright American Society of Nuclear Cardiology
